# The Role of Digital Tools in the Timely Diagnosis and Prevention of Acute Exacerbations of COPD: A Comprehensive Review of the Literature

**DOI:** 10.3390/diagnostics12020269

**Published:** 2022-01-21

**Authors:** Athanasios Konstantinidis, Christos Kyriakopoulos, Georgios Ntritsos, Nikolaos Giannakeas, Konstantinos I. Gourgoulianis, Konstantinos Kostikas, Athena Gogali

**Affiliations:** 1 Respiratory Medicine Department, Faculty of Medicine, University of Ioannina, 45500 Ioannina, Greece; akonstan@uoi.gr (A.K.); ktkostikas@gmail.com (K.K.); athenagogali@yahoo.com (A.G.); 2 Department of Hygiene and Epidemiology, Faculty of Medicine, University of Ioannina, 45500 Ioannina, Greece; gntritsos@uoi.gr; 3 Department of Informatics and Telecommunications, School of Informatics and Telecommunications, University of Ioannina, 47100 Arta, Greece; giannakeas@uoi.gr; 4 Department of Respiratory Medicine, Faculty of Medicine, University of Thessaly, 41110 Larissa, Greece; kgourg@med.uth.gr

**Keywords:** telemedicine, telehealth, telemonitoring, COPD, acute exacerbation COPD, diagnosis, prevention

## Abstract

Chronic obstructive pulmonary disease (COPD) is a chronic inflammatory disease of the airways and lung parenchyma with multiple systemic manifestations. Exacerbations of COPD are important events during the course of the disease, as they are associated with increased mortality, severe impairment of health-related quality of life, accelerated decline in lung function, significant reduction in physical activity, and substantial economic burden. Telemedicine is the use of communication technologies to transmit medical data over short or long distances and to deliver healthcare services. The need to limit in-person appointments during the COVID-19 pandemic has caused a rapid increase in telemedicine services. In the present review of the literature covering published randomized controlled trials reporting results regarding the use of digital tools in acute exacerbations of COPD, we attempt to clarify the effectiveness of telemedicine for identifying, preventing, and reducing COPD exacerbations and improving other clinically relevant outcomes, while describing in detail the specific telemedicine interventions used.

## 1. Introduction

Chronic obstructive pulmonary disease (COPD) is a chronic debilitating disease of the airways and lung parenchyma with a prevalence of approximately 380 million cases worldwide [1]. It is currently the third leading cause of death, responsible for approximately 6% of the world’s total deaths (approximately 3.3 million annually) [2]. An exacerbation of COPD (AECOPD) is defined as an acute worsening of respiratory symptoms requiring additional therapy, usually caused by a viral or bacterial lung infection [3]. AECOPDs are important events during the course of the disease because they are associated with increased mortality, severe impairment of health-related quality of life, accelerated decline in lung function, significant reduction in physical activity, and substantial economic burden [4,5,6].

**Figure 1:**
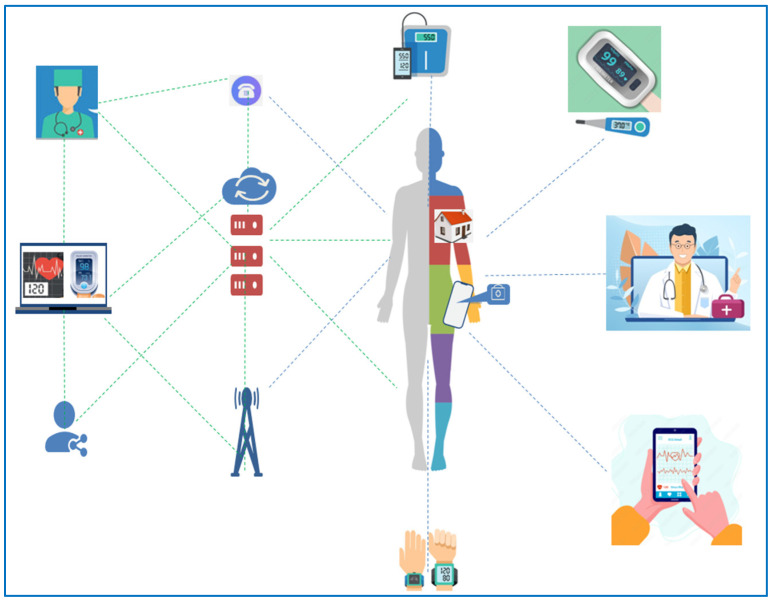
The telemedicine ecosystem.

Telemedicine is defined as the use of communication technologies to transmit medical data over long and short distances and to deliver healthcare services [7]. The term telemedicine (from the Greek term ‘‘tele’’ and the Latin ‘‘medicus’’) was devised in the 1970s by the American Thomas Bird and actually means ‘‘treating from a distance’’ [8]. The origins of telemedicine date back to 1905, when Willem Einthoven successfully transmitted both the first electrocardiogram and the heart sounds of a volunteer between his lab and the Academic Hospital in Leiden, the Netherlands by a telephone line [9]. Progress in telemedicine has been expedited due to recent technological advances that offer user-friendly and reliable applications, as depicted in Figure 1. In addition, the need to limit in-person appointments during the COVID-19 pandemic has caused a rapid increase in telemedicine services.

Telemedicine applications in COPD were introduced more than 20 years ago but have rapidly expanded over the last decade [10]. These applications include tracking COPD patients for earlier detection of disease exacerbations and early intervention to prevent deterioration and the need for hospitalization [11]. Given that exacerbations of COPD are associated with high mortality and morbidity as well as substantial healthcare expenditures, well-designed telemedicine applications might become a valuable tool for reducing AECOPDs.

A number of systematic reviews and meta-analyses have evaluated the role of telemedicine in various clinical outcomes in patients with COPD [7,12,13,14,15,16,17]. A recent systematic review and meta-analysis of 22 randomized controlled trials (RCTs) involving 2906 participants in telemonitoring (TM) interventions for severe COPD exacerbations suggested that the addition of TM to usual care decreased avoidable emergency department (ED) visits but was unlikely to prevent hospitalizations due to COPD exacerbations [18]. However, the authors underline the fact that there was ‘high’ bias in the ‘blinding of participants and personnel’ in the included studies because it might have been difficult for the participants and personnel to remain unaware of the interventions due to the nature of TM interventions. In addition, they comment that there was significant clinical heterogeneity between trials in terms of the study duration, study population, patient recruitment setting, type of technology used, and TM interventions [18]. Similar diversity in outcomes has been found among meta-analyses involving various telemedicine interventions in patients with COPD, attributed to the complexity of telemedicine applications, lack of validated data collection instruments, and lack of high-quality reporting [11].

The evidence as to whether digital tools are actually effective in early detection and prevention of AECOPDs seems inconclusive and contradictory. In our comprehensive review of the literature, we attempt to clarify the effectiveness of telemedicine for the identification, prevention or reduction in AECOPDs. Specifically, we aim to classify the studies into those reporting significant improvement in outcomes and those reporting non-significant ones, and to describe in detail the specific telemedicine interventions used in each study.

## 2. Methods

We conducted a search of EMBASE, PubMed, and Scopus databases using the following search algorithm (“chronic obstructive pulmonary disease” OR “chronic obstructive airway disease” OR “chronic obstructive lung disease” OR “chronic obstructive bronchitis” OR “COPD”) AND (“telemonitoring” OR “telehealth” OR “telemedicine” OR “telecommunication” OR “remote consultation”) AND (“random” OR “trial” OR “randomised controlled trial” OR “randomized controlled trial” OR “clinical trial” OR “RCT”). Papers published between 2006 and October 2021 in the English language were considered. C. K. and A. G. assessed the identified randomized controlled trials (RCTs) studies for suitability. Among the 325 identified studies, 35 RCTs containing a TM and control group reported results regarding digital tools in AECOPDs and were considered appropriate for inclusion in the review.

## 3. Studies with Positive Results

### 3.1. Telemedicine Involving Close Healthcare Monitoring

An RCT conducted in Taiwan by Ho et al. [19] including 106 COPD patients showed a beneficial effect of TM in preventing AECOPDs and related admissions, using an electronic diary for the intervention group, which was easy to fill, consisting of 8 questions about symptoms, vital signs, and weight. Based on an algorithm, data were scored and transmitted to the medical team, while warnings related to the scores achieved generated notifications if appropriate. TM took place for 2 months, with atotal follow-up of 6 months, while the control group received the usual care. The time to first readmission for AECOPD was increased in the TM group as compared with the usual care group (*p* = 0.026). The probability of COPD-related readmission was significantly lower in the TM group (hazard ratio 0.42, 95% CI: 0.19–0.92), and there was a trend for fewer episodes of COPD-related readmissions (0.19 vs. 0.49; *p* = 0.11) or ED visits (0.23 vs. 0.55; *p* = 0.16) in this group. The first COPD-related ED visit was also delayed (hazard ratio = 0.50). Finally, TM intervention was associated with significant reductions in the numbers of all-cause readmissions and ED visits for all causes compared to the usual care group. Investigators attributed the effectiveness of TM to the items monitored and corresponding algorithm [19].

Kessler et al. [20] included 319 COPD patients with a mean forced expiratory volume in one second (FEV1) of 37.1% predicted and at least one severe exacerbation in the previous year. Patients in the telehealth (TH) group were under home monitoring and an e-health telephone and web platform was applied that transmitted FEV1, heart rate (HR), pulse arterial oxygen saturation (SPO_2_) plus daily oxygen use, and respiratory ratio (RR) data for patients on long-term oxygen therapy (LTOT). Upon deterioration, patients were contacted by the investigators. No significant difference was observed regarding hospitalization days, hospital admissions, AECOPDs, six minute walking test (6MWT), Saint George’s respiratory questionnaire (SGRQ), or hospital anxiety and depression scale (HADS) scores. Nevertheless in the TH group, acute care hospitalizations and mortality rate were significantly lower (1.9% vs. 14.2%, *p* < 0.001), with greater improvements in body mass index, airflow obstruction, dyspnea, and exercise capacity (BODE) index score and a higher proportion of patients who quit smoking. One possible explanation for the mortality rate reduction is that the TH management program may have promoted earlier intervention, thereby preventing fatal complications of AECOPDs [20].

Koff et al. [21] recruited 40 GOLD COPD stage III or IV patients, administering in the TM group a pulse oximeter, a FEV1 monitor, a pedometer, and a technology platform for delivery of education and transmission of the results for 3 months. When a clinical problem emerged, the coordinator would help facilitate its resolution by providing the appropriate instructions. Patients receiving integrated care demonstrated a lower number of hospitalizations and ED visits, improvement of SGRQ, and reduced healthcare costs. However, the generalizability of the results is limited by the small number of participants and the short duration of the study [21].

Pedone et al. [22], in an RCT including elderly patients with COPD in stages II and III, used a simple, fully automated system that required no effort on the patient’s side and was able to monitor vital signs several times a day and transmit them to a skilled physician that could contact and advise the patient when appropriate. In total, 50 patients received the intervention and 49 received usual care, who were followed for 9 months. Although statistical significance was not reached due to the lower incidence of events than expected, a beneficial effect of home telemonitoring was shown, with a 33% reduction in exacerbations (incidence rate ratio 0.67, 95% CI: 0.32–1.36) and related admissions (incidence rate ratio 0.66, 95% CI: 0.21–1.86). The only parameter monitored able to identify timely AECOPD was oxygen saturation. Surprisingly, the average length of stay was shorter for the control group, possibly because TM helped patients dealing with less severe exacerbations at home [22].

Similarly, Segrelles-Calvo et al. [23] recruited 60 COPD GOLD stage III–IV patients on LTOT in order to assess the efficacy and effectiveness of a home TH program for COPD patients with severe or very severe airflow obstruction. TM parameters included oxygen saturation, HR, and blood pressure (BP) daily and peak expiratory flow (PEF) results three times a week. In the TH group, reductions in the number of ED visits, hospital admissions, length of hospitalization, and mortality were observed. The positive results of this study could be attributed to the combination of TH resources with conventional care and prompt interventions after an early detection of an AECOPD, together with the coordination of primary care, pneumologists, and nursing staff. Moreover, the accessibility of the TM device and the overall satisfaction rate, which was high, led to no withdrawals [23].

Shanny et al. [24] assessed the effects of TM in 42 COPD patients with severe or very severe airflow obstruction. The TH intervention included measurements of pulse saturation, temperature, HR, BP, and weight, daily symptoms, electrocardiogram, and spirometry, with telephone support and home visits. Reductions in the total length of stay for all admissions, time to first hospitalization, relative risk of ED visit or hospital admission, and hospitalization costs were observed, while there was also a trend towards reductions in the numbers of ED visits and hospital admissions [24].

In an RCT with a different design, Sink et al. [25] recruited 168 COPD patients with mild to very severe airflow obstruction. Patients allocated to the treatment group received a daily message regarding their breathing status. If a patient responded “worse”, then the EpxCOPD system immediately triggered an alert to the assigned medical resident provider, while if they were experiencing a medical emergency, they were advised to present to the emergency department to seek care. The time to hospitalization was significantly different between the two groups, favoring the TM group (hazard ratio 2.36, 95% CI: 1.02–5.45; *p* = 0.043), while the same applied for the number of hospital admissions. The positive results were associated with the early intervention and proper consultation by the health staff, leading to the prompt detection and treatment of AECOPDs and finally to the reduction in hospital admissions [25].

Vitacca et al. [26] studied 101 COPD patients with the need for home mechanical ventilation (HMV) or LTOT and at least one hospitalization for AECOPD in the previous year, with a mean FEV1 of 39% pred. Patients allocated to the TH group received a pulse oximetry device that transmitted pulse saturation data via a telephone modem to a receiving station, where a nurse was available to provide a real-time teleconsultation, for 12 months. The on-duty respiratory physician was informed for unscheduled calls and provided a consultation. The study demonstrated that patients in the intervention group had fewer hospital admissions per month compared with controls (mean (SD) 0.17 (0.23) vs. 0.30 (0.30); *p* = 0.019), experienced fewer hospitalizations (*p* = 0.018), and had a higher probability of avoiding hospitalization (*p* = 0.012). Moreover, patients in the TM group had a significantly higher probability of remaining free from AECOPDs (*p* = 0.0001), from further urgent general practitioner (GP) calls (*p* = 0.013), and from further ED visits (*p* = 0.003). However, the mortality rates did not differ between the two groups (*p* = 0.148) [26].

Clemente et al. [27] performed a randomized clinical trial to examine the role of telemedicine in monitoring early-discharged and home-hospitalized COPD patients after an exacerbation. The intervention group underwent TM during home hospitalization using a multiparametric recording unit. Data on vital constants (electrocardiogram (ECG), SPO2, HR, BP, temperature, and RR) were transmitted twice per day to the physician in charge with a subsequent phone call by him concerning their clinical situation, and only 2 home visits by healthcare staff were performed (intermediate and at discharge). The control group received daily visits by nursing staff and a final visit from the physician. Both groups were then followed for six months without TM. The main outcome was time until first exacerbation; no difference was observed, with a median of 48 days in the control group and 47 days in the intervention group (*p* = 0.52). Additionally, during the follow-up period, no significant difference was observed in the numbers of AECOPDs that both groups experienced. Notably, the durations of home hospitalization were similar in the two groups (median 7 days), as were the numbers of readmissions observed. Thus, in this study, home hospitalization using telemedicine after early discharge proved non-inferior to conventional home follow-up [27].

A different aspect of how telemedicine can improve the health of COPD patients is given by the study of de Toledo et al. [28], who used a technological platform called the Chronic Care Management Center, which consists of a telephone center and a telemedicine server that gives access to the electronic chronic patient records to all the members of the care team from any location (patient’s home, hospital, primary care center). The study lasted for a year and a total of 157 COPD patients were recruited during hospital admission due to an exacerbation. The intervention group had telephone access to the system’s center, while the multidisciplinary team caring for these patients had access to the platform. Follow-up of the control group did not involve phone calls or telemedicine support for the health providers. Care coordination and telephone support to patients led to a significant reduction in readmissions (number of patients that did not need a readmission: 51% intervention vs. 33% control; *p* = 0.04), possibly due to early detection of AECOPD symptoms. No difference in ED visit number or mortality was demonstrated [28].

### 3.2. Telemedicine Involving Primarily Self-Management Techniques

Casas et al. [29] showed that integrated care intervention could prevent COPD-related hospitalizations. Here, 155 COPD patients from two different centers, Barcelona and Leuven, who had an exacerbation, were recruited immediately after discharge, and 65 of them were offered integrated care, which consisted of an individual well-defined care plan shared between the primary care and hospital team, as well as accessibility to a specialized nurse through a web-based call center. Patients in the intervention arm were educated before discharge on several issues, including self-management techniques, receiving an early joint visit from the specialized nurse and the primary care team or regular visits from their GP, who had been contacted by the primary investigator. They also received weekly reinforcement calls from the specialized nurse in the first month, while a chronic platform (a call center coupled to a web-based application providing access to the patients’ records) was accessible by them and primary care providers. Patients in the control arm were visited by their own physician, usually every six months, without additional support. During 12-month follow-up, a significantly lower readmission rate was observed in the intervention group (hazard ratio 0.55, 95% CI: 0.35–0.88; *p* = 0.01), the percentage of patients without admissions was greater, while the difference in the rate of admissions per patient between the follow-up and the previous year was also lower. No survival differences were found. This positive result was possibly due to a combination of the effectiveness of patient education programs along with the personalized health plan and higher accessibility to healthcare professionals [29].

In a different approach, Jehn et al. [30] monitored 32 patients specifically during the summer period (9 months in total), showing that climate change (heat stress) has a negative impact on the clinical status, lung function, and exercise capacity of COPD stage II–IV patients. Interesting remarks were made regarding exacerbations when the TM group was compared to the control group. More specifically, the TM intervention included a daily COPD assessment test (CAT), spirometry, and a weekly 6MWT measured by accelerometry performed at home. Data were transmitted and reviewed daily, although the study had an observational character. The intervention group exhibited significantly fewer AECOPDs during summer (3 for TM vs. 14 for control group; *p* = 0.006) and over the rest of the year. Over the whole 9-month follow-up period, the intervention group had a significantly lower number of exacerbation-related hospital admissions (7 vs. 22; *p* = 0.012), spent significantly less time in hospital due to COPD (34 vs. 97 days), and performed significantly fewer visits to the pulmonologist (24 vs. 42; *p* = 0.042). As there had been no medical intervention, positive results were attributed to better disease awareness and improved physical condition, possibly due to weekly performance of the 6MWT [30].

Paré et al. [31] included 120 COPD patients with a FEV1 under 45% pred. and at least one hospitalization in the previous year. A digital device was provided to the intervention group and the patients had to complete a daily data entry table documenting symptoms and adherence to prescribed medication for 6 months. Patients were afterwards under surveillance for the next 6 months, after the initial 6-month period. During the initial 6-month period, the TM group exhibited less ED visits (36% vs. 13% reduction, without statistical significance) and hospital admissions, shorter length of hospitalization, less home visits by nurses, and lower healthcare costs [31].

### 3.3. Telemedicine Involving Telerehabilitation

Dinesen et al. [32] constructed an RCT that showed that telerehabilitation using a TM device could significantly reduce hospital admissions in patients with COPD. In total, 111 stage III and IV patients were randomized and 60 of them were instructed to use digital equipment in order to measure, monitor, and transmit vital signs, training inputs of the rehabilitation program, and spirometry values. All healthcare professionals (GP, nurses, and hospital doctors), patients, and relatives had access to the data, while once a month a video meeting was held between healthcare teams to coordinate the individual rehabilitation programs of the patients. The control group performed home exercises by themselves, without any contact. The TM program had a duration of 4 months and patients were followed-up for 10 months in total, resulting in a reduction in admission rate to 0.48 over 10 months compared to 1.17 for the control group (*p* = 0.041). A trend towards a longer time to first admission was also observed in the telerehabilitation group. A positive preventative impact of the study was attributed to the improved self-management of the illness and interactions among healthcare professionals and the patients with the help of technology [32].

Tabak et al. [33] performed a pilot RCT including 29 COPD patients with three or more AECOPDs or one hospitalization for respiratory disease in the preceding 2 years. Patients in the TH group received a web-based exercise program, an activity coach, a self-management module, and a teleconsultation module on the web portal for 9 months. The TH group showed a lower number of hospital admissions, shorter hospitalization stay, and improvement in the quality of life satisfaction with received care. These findings are limited in their generalizability by the fact that it was a pilot study, the small number of participants, and the significantly worse dyspnea levels in the control group [33].

Vasilopoulou et al. [34] recruited 147 GOLD COPD stage II or IV patients with at least one AECOPD in the year prior to the study. Following the completion of an initial 2-month pulmonary rehabilitation program, this RCT compared 12 months of home-based maintenance telerehabilitation (*n* = 47) with 12 months of hospital-based outpatient maintenance rehabilitation (*n* = 50) and also 12 months of usual care treatment (*n* = 50), without initial pulmonary rehabilitation. Patients in the TM group received an oximeter, a spirometer, a pedometer, and a video demonstration of the home exercises. The TH intervention led to lower rates of AECOPDs, hospital admissions, and ED visits; improvement of health-related quality of life (HRQL); higher functional capacity; and higher daily physical activity compared to the usual care group. Notably, the results were comparable with the hospital-based rehabilitation program for all parameters, while the reduction in ED visits was even greater in the telerehabilitation arm [34]. Table 1 summarizes the characteristics of the RCTs for TM, which showed positive results.

## 4. Studies with Negative Results

### 4.1. Telemedicine Involving Primarily Close Healthcare Monitoring

Antoniades et al. [35], in a pilot study including 44 patients with moderate to severe COPD, aimed to explore the feasibility of remote monitoring and a possible reduction in healthcare use when this intervention is added to the standard of care (SOC). Multiple parameters were monitored daily over a 12-month period (spirometry, weight, temperature, BP, SPO2, ECG, sputum color and volume, symptoms, and medication usage) with close revision by a nurse and frequent reminders. Adherence of up to 80% was observed, although the addition of TM did not achieve a significant reduction in admission rate (SOC 1.5 ± 1.8 vs. TM 1.3 ± 1.7, *p* = 0.76) or length of hospitalization (SOC 15.6 ± 19.4 vs. TM 11.4 ± 19.6, *p* = 0.66). Possible explanations are that the SOC was not usual but was a very high-quality multidisciplinary referral institution approach to COPD management, as well as the small size of the study. Nevertheless, patients who received TM exhibited a significant trend toward fewer admissions during the study period compared with the previous year (1 (0–2) admission/year during study period compared to 2 (1–4) admissions/year during the year before, *p* = 0.052), implying that matching in terms of history of prior admissions instead of disease severity and smoking habit may have altered conclusions [35].

Boer et al. [36] recruited 87 patients with two or more AECOPDs, comparing exacerbation self-management strategies using either a paper exacerbation action plan (control group) or an innovative mobile health tool (mHealth group), consisting of a mobile phone, a pulse oximeter, a spirometer, a thermometer, and a question–answer system, which provided automated advice based on a decision tree manufactured by experts and with the help of a Bayesian prediction model (intervention group). In terms of exacerbation-related outcomes, no differences were found between the two groups in the number of exacerbation-free weeks, in the number of symptom-based exacerbations, the exacerbations treated with antibiotics or prednisone, the unscheduled healthcare contacts, or the hospital admissions. Furthermore, no difference in timely action that would prevent an exacerbation was identified between the two groups. Apart from the small size of the study, this result could be explained by the small room of improvement left, as both groups were provided an introductive education session based on the recognition and treatment of exacerbations [36].

Chau et al. [37] conducted a small 8-week study including 40 older COPD patients with moderate or severe disease and history of at least one hospital admission for exacerbation in the past year. Twenty-two patients in the intervention group received a telecare device kit that monitored SPO2, HR, and RR indices transmitted to a health provider via an online platform that could act promptly. A high level of user satisfaction was reported but no significant differences in the numbers of ED visits and hospital readmissions between the groups were observed. Nevertheless, the very small duration and size of the study did not permit extraction of safe conclusions [37].

Cordova et al. [38] focused their research on daily symptom reporting using telemedicine in a 24-month randomized control trial with 79 participants that had been hospitalized for an AECOPD within the past year or were using supplemental O_2_. While the 40 patients in the control group were provided with peak flow meters and electronic diaries for symptom reporting and had been advised to seek medical assistance if they worsened, the 39 patients in the intervention group received a telecommunication device for symptom reporting and scoring via a computerized algorithm that included a score “alert”. The alert was generated when the symptom score reflected a significant alteration of the baseline symptoms, initiating appropriate medical intervention that was available 24 h/day. The symptom assessment included PEF; dyspnea; and sputum quantity, color, and consistency. Coughing, wheezing, sore throat, nasal congestion, and temperature were considered minor symptoms. Although there were no differences in hospitalization rates, hospitalization durations, times to first hospitalization, or mortality rates between groups, acute exacerbation symptoms were significantly different, with the intervention group reporting significantly fewer moderate and severe symptom days (*p* < 0.0001). The failure to show either a mortality benefit or reduction in hospitalization days was attributed by the authors to the slow recruitment rate and to a lower hospitalization rate observed across both groups due to the optimized care [38].

De San Miguel and colleagues [39] attempted to show that self-monitoring combined with remote monitoring via a TH instrument that measured COPD patients’ vital signs could make a difference in health service utilization. In this RCT, which had a duration of 6 months and included 80 patients, BP, weight, temperature, HR, and oxygen saturation levels were measured and transmitted automatically to the Internet, along with patients’ daily answers related to their health, which became available to the TH nurse daily. Information triggered an intervention when appropriate, while the parameters were also made available to each participant’s physician. The intervention group had fewer hospitalizations and ED presentations (almost half), as well as a reduced length of in-hospital stay (77 fewer days in total), with a significant impact on health costs. However, the study did not reach statistical significance, possibly due to the fact that it was performed in the summer period, when the hospital admission rate was lower than expected [39].

Jakobsen et al. [40] compared home-based TH hospitalization in patients with severe COPD that were admitted with an acute exacerbation and had an expected hospitalization of more than 2 days to standard in-hospital treatment. Patients needing non-invasive or invasive mechanical ventilation or intravenous antibiotics, had pH < 7.35, or with serious comorbidities were excluded. This was a non-inferiority study and treatment failure was defined as readmission due to COPD within 30 days after discharge. The intervention group (29 patients) were provided with a touch screen with a webcam, pulse oximeter, spirometer, thermometer, and medicines, and participated in daily scheduled virtual ward rounds, while acute contacts were possible through a “call hospital” button. The control group (28 patients) was hospitalized as usual. The study did not meet the primary endpoint, which was a treatment failure rate less than 20% higher than that of the control group. A possible explanation for this is the small sample size. No participants died within 30 days of the discharge and 3 patients returned to the hospital due to technical failure, hyponatremia, and nebulizer failure [40].

Another Spanish study by Jodar-Sanchez et al. [41], including 45 GOLD stage III–IV patients with at least one severe exacerbation in the previous year, showed no benefit from TM in terms of preventing serious exacerbations. The investigators included patients with advanced COPD treated with long-term oxygen therapy. The intervention group measured daily vital signs and spirometry twice a week, while transmitted measurements were followed and evaluated and clinical responses were generated by alerts. Although a small, non-significant reduction was observed in the number of ED visits, hospital admissions were not fewer and most of the patients admitted had previously visited the ED. It was noted that 33% of the serious exacerbations leading to hospitalization occurred during weekends and holidays when the TH program was interrupted, a fact that should be taken into consideration in future studies [41].

McDowell et al. [42] recruited 110 GOLD stage II–III patients with at least two ED admissions, hospital admissions, or emergency GP contacts in the 12 months before the study. The TM device recorded BP, HR, and SPO2 for six months and patients answered questions relating to symptoms (difficulty in breathing, cough, sputum, tiredness). If an alert occurred, a nurse contacted the patient and provided advice; if further escalation was necessary, the nurse contacted the respiratory physician, who decided whether a home visit or an ED admission was required. There were no significant differences between the two groups in exacerbations, hospital admissions, ED visits, total GP calls, EQ-5D quality of life scores, or HADS depression scores, and the intervention was not cost-effective. However, the SGRQ score improved significantly in the TM group compared to usual care, exceeding the minimum clinically important difference of at least four units (mean difference 5.75, 95% CI: 2.32–9.18; *p* = 0.001), and the same applied for the HADS anxiety score (*p* = 0.01). The negative results regarding AECOPDs, hospital admissions, ED visits, and total GP calls could be attributed to the short duration of the TM (6 months) [42].

Pinnock et al. [43] included 256 COPD patients with admission to the hospital with an AECOPD in the previous year. Patients in the TM group completed a daily questionnaire regarding symptoms and use of treatment, and SPO2 was monitored. For the symptom scores, the patients were asked to assess whether their dyspnea, cough, wheeze sputum purulence, and volume had increased and if they had a fever or had developed an upper respiratory tract infection. No significant difference was observed in the time to first hospitalization, time to first hospitalization with an AECOPD or all cause death, number and duration of hospital admissions with an AECOPD, number and duration of admissions for any cause, number of exacerbations self-reported by participants, HRQL, anxiety, or depression. However, patients in the TM group exhibited a significantly lower number of deaths and higher use of healthcare resources [43].

Rose et al. [44] evaluated the effectiveness of a multi-component intervention including individualized care action plans and telephone consults (12-weekly then 9-monthly) for reducing emergency department visits. In total, 470 COPD patients with one or more ED visits or hospital admissions for AECOPD in the previous 12 months and two or more prognostically important COPD-associated comorbidities were randomized. The TH intervention had no impact on either the number of ED visits or the number of hospital admissions. However, TH significantly reduced mortality, a finding that could not be fully explained by the authors [44].

Rinbaek et al. [45] recruited 281 COPD GOLD stage III–IV patients with one hospital admission due to AECOPD within the previous 36 months or treated with LTOT for at least 3 months. Patients allocated to the TM group were provided a tablet computer with a web camera, a microphone, and measurement equipment (spirometer, pulse oximeter, and mMRC scale), while patients reported changes in dyspnea and sputum color, volume, and purulence. There was no significant difference between the two groups in number of hospitalizations, AECOPDs, ED visits, length of hospitalization, all-cause hospital admissions, time to first hospital admission, or all-cause mortality. Patients in the TM group demonstrated a significantly lower number of visits to the outpatient clinic and a significantly higher number of AECOPDs requiring treatment with systemic steroids and antibiotics but not admission to hospital. The negative results of the study could be attributed to the short duration of the TM (6 months) [45].

In a similar RCT, Soriano et al. [46] recruited 229 COPD GOLD stage III–IV patients with LTOT and two or more moderate or severe AECOPDs in the previous year, with or without hospitalization. Patients in the TM group received a pulse oximeter, a blood pressure gauge, a spirometer, a respiratory rate, and an oxygen therapy compliance monitor connected to the oxygen feed from their main oxygen source. No significant difference in number of AECOPDs, ED visits, hospital admissions, mortality, health related costs, or quality of life was observed. However, there was a trend towards shorter lengths of hospitalization and days in intensive care unit. The negative results of the study could be attributed to the fact that patients in the TM group had more hospital admissions in the previous year, the reduced primary care integration (since it was a multicenter study), and the high sensitivity of the clinical alert threshold applied to the TH group, which may have increased ED visits and hospital admissions due to false alerts [46].

Vianello et al. [47] recruited 315 COPD GOLD stage III–IV patients with a mean FEV1 of 41.9% pred. at the time of discharge from hospital after an AECOPD episode or while attending the outpatient respiratory medicine clinic. A finger pulse oximeter and a gateway device for data transmission over a telephone line to a central data management unit were applied to the patients for a 12-months period. When the transmitted values were considered out-of-range, the clinical staff were alerted and the patient was contacted. The readmission rate for AECOPD was significantly lower in the TM group compared to the standard care group (incidence rate ratio 0.43, 95% CI: 0.19–0.98; *p* = 0.01), and the same applied for the number of appointments with a pulmonary specialist (incidence rate ratio 0.82, 95% CI: 0.67–1; *p* = 0.049). However, the number and duration of hospitalizations due to AECOPD or any other cause, the number of ED visits, and the number of deaths did not differ significantly between the two groups, and neither did TH improve the quality of life or emotional distress. The possible explanations for the negative results were firstly the very low number of hospital admissions, which did not leave enough space for further reductions as a result of a TM intervention, and secondly that changes in HR and SPO2, which are used as markers of an unstable clinical condition, are often unable to reflect changes in patient health status, leading to underestimation of AECOPDs [47].

Walker et al. [48] included 312 patients with COPD GOLD stage II or higher and a history of AECOPD, with or without hospitalization in the previous 12 months, from 5 European countries, applying the CHROMED monitoring platform for 9 months at approximately the same time each day. The platform comprised a device that measured within-breath respiratory mechanical impedance using the forced oscillation technique, a touch screen computer, and a mobile modem. When an alert was generated, a variety of actions were possible, ranging from no action to taking a course of antibiotics or corticosteroids or face-to-face assessment. There was no difference between the two groups in time to first hospitalization, number of hospitalizations, number of moderate exacerbations, or number of patients free from hospital admission. However, among patients previously hospitalized for AECOPD, there was a 53% reduction in the hospitalization rate in the treatment group compared with the control group (0.85 vs. 1.88 admissions/year; *p* = 0.017). The negative results of the study could be attributed to the low number of hospitalizations and the variation between different healthcare systems [48].

Telemonitoring requires commitment and appropriate training of the study team in order to help COPD patients and to extract safe conclusions regarding the intervention’s role in preventing exacerbations. This was shown in a pilot study by Bentley et al. [49], which demonstrated a negative impact of TM when comparing to face-to-face visits. The SOC team received 6 home visits after discharge, while the intervention team used TH equipment that could monitor and transmit vital signs to the study clinicians after the third visit. The intervention lasted 8 weeks. An increase in hospital admissions (34% vs. 16%) and a higher duration of hospitalization were reported during the 8-month follow-up period. The study had serious issues, such as slow recruitment and gaps in data collection due to problems related to research team dedication (a frontline clinical team who experienced a 60% loss of staff capacity during the study) and inadequate staff training [49].

### 4.2. Telemedicine Involving Primarily Self-Management Techniques

Self-management supported by a digital health system did not improve exacerbation-related outcomes in the study by Farmer et al. [50]. A fully automated, Internet-linked, tablet-computer-based system for monitoring and self-management support was provided to 110 patients with moderate to very severe COPD, while 56 patients received the usual care. The intervention consisted of the monitoring of oxygen saturation and HR; a daily symptom diary with questions regarding general well-being, cough, breathlessness, sputum, and use of medications; monthly mood screening questionnaires; and training videos with inhaler techniques, pulmonary rehabilitation exercises, and self-management techniques for breathlessness. Safety thresholds for vital signs, symptoms, or psychological scores generated “alerts” and data were reviewed by a member of the respiratory team twice weekly. The study duration was 12 months. The primary outcome related to quality of life improvement was not achieved and no reduction in the number of exacerbations was seen in the intervention group. The relative risk of hospital admission for the digital health group was 0.83 (0.56–1.24, *p* = 0.37) and no reduction in the use of healthcare services was noticed, except for fewer visits to the GP practice nurses (1.5 for digital health versus 2.5 for usual care, *p* = 0.03) [50].

Lewis et al. [51] included 40 COPD GOLD stage II–III patients after completing at least 12 sessions of pulmonary rehabilitation. In the TH group, oxygen saturation, temperature, and symptoms were recorded daily for 26 weeks. There was no difference between the two groups in ED visits, hospital admissions, or hospitalization days during the monitoring period; however, in the TM group, fewer primary care contacts for chest problems (*p* < 0.03) were documented. The small size of the study, the short duration, and the fact the participants in the TM group were stabilized, since they had completed the pulmonary rehabilitation course, could have contributed to the negative results of the study [51].

Rassouli et al. [52] recruited 168 COPD patients with a mean FEV1 of 51%. Patients in the TH group completed CAT scores weekly and answered six questions focused on the detection of AECOPD daily. There was no difference in favor of the TH group regarding total number of AECOPDs, hospital admissions, or ED visits. Nevertheless, in the TH group, the rate of CAT increase was significantly reduced, more moderate AECOPDs were detected, the satisfaction with care was higher, and there was a trend towards shorter length of hospitalization and reduced COPD-related costs. The negative results regarding AECOPD outcomes resulted from the fact that the study’s primary outcome was difference in weekly CAT score, so there was insufficient statistical power to show significant differences, which could be achieved by including considerably more patients [52].

In an RCT with a different approach, Sorknaes et al. [53] evaluated the effects of 7 days real-time teleconsultations between hospital-based nurses and patients discharged after hospital admission for an AECOPD. The study included a total of 266 COPD patients discharged after an AECOPD. Patients in the TM group received a video device, an oximeter, and a spirometer; they were evaluated daily for one week and received a proper teleconsultation from a nurse, initiated 24 h after discharge and with a 26-week follow-up. No significant difference in total hospital readmissions, time before first readmission, mortality, time to mortality, hospital readmissions per patient, or hospital days per patient at 4, 8, 12, or 26 weeks after discharge was observed. The negative results could be attributed to the very short duration of the intervention (7 days) and to the fact that the teleconsultation was provided only by a nurse through a checklist [53]. Table 2 summarizes the characteristics of the RCTs on TM that showed negative results.

## 5. Conclusions and the Way Forward

The goal of telemedicine is the early identification and diagnosis of AECOPDs and timely access to appropriate treatment in order to improve patient outcomes. An important issue for the effective application of telemonitoring in COPD is the parameters monitored in terms of reliable prediction of an AECOPD (e.g., FEV1, symptoms, pulse oximetry). When an algorithm is used, the design is even more crucial regarding the satisfactory sensitivity and specificity of the method. Moreover, the effort that was required from the patients was not associated with the outcomes of the studies; however, it is likely that patients would be more compliant to interventions that would require minimal effort on their end. The results of the negative studies regarding exacerbations, hospital admissions, ED visits, and total GP calls could be attributed to the short telemonitoring period and the fact that in most of them, healthcare utilization and exacerbations were not the primary endpoints. Importantly, the small number of participants may have led to negative results and may have additionally precluded the extraction of safe conclusions. Additionally, the short follow-up periods of many studies were not long enough to capture the natural history of AECOPD, and in our opinion a minimum of 12 months would be required. Moreover, adherence to inhaled medication, which is enhanced during RCTs, may additionally lead to reduced AECOPDs and hospital admissions in both intervention and usual care groups, thereby resulting in non-significant differences. The commitment and appropriate training of the study team involved in such programs is crucial for successful telehealth services, while the interruption of monitoring during weekends by healthcare interventions may have affected the results in some cases, although prevention of exacerbations was reported without active intervention by the study team, which was attributed to better disease awareness and self-management. Finally, it is unknown which patients will benefit more from telemedicine, and larger RCTs are needed with subgroup analysis to define the most appropriate population for telemonitoring interventions. These studies would need to be of a proper length and size, and following a multifactorial evaluation would need to involve the appropriate participants who will adopt these telemonitoring interventions, which ideally will require minimal patient effort, in order to maximize the engagement and potential benefits. The proposed characteristics of future studies are summarized in Table 3.

## Figures and Tables

**Table 1:** Telemedicine studies showing positive results.

Author (Year)	Country	Primary Objective	Secondary Objectives	COPD Severity	TM Duration	*n* Telemonitoring Group	*n* Control Group	Patient Effort Required	Telemonitoring Intervention	Telemonitoring Data	Exacerbation Outcomes	Other Study Outcomes
Casas (2006) [29]	Spain, Belgium	Rehospitalization rate, mortality	-	Discharge after AECOPD	12 m	65	90	Mild	Web-based call center	-	Readmission rate-	Mortality=
De Toledo (2006) [28]	Spain	Readmissions, ED visits, mortality	Acceptability to professionals, characterization of the patterns of use of the system, costs	Discharge after AECOPD	12 m	67	90	Mild	Chronic care telemedicine system, phone calls by patients	Electronic chronic patient record accessible to the care team	Readmissions-; ED visits=; mortality=	Acceptability+
Koff (2009) [21]	USA	HRQL	AECOPDs, healthcare costs	COPD GOLD stage III–IV	3 m	20	20	High	Telemonitoring plus self-management plus phone contact	PFTs, SPO2, 6MWT, shortness of breath, cough	Hospital admissions-; ER visits-	SGRQ-; costs-
Vitacca (2009) [26]	Italy	Reduction in hospital admissions	Reduction in AECOPDs, ED visits, urgent GP calls, cost-effectiveness	Need for HMV and/or need of LTOT and at least one hospitalization for AECOPD in the previous year, FEV1 % pred. 39%	12 m	57	44	Mild	Telemonitoring plus telenursing and doctor on demand	SPO2	Hospital admissions-; AECOPDs-; urgent GP calls-; mortality=	Cost-effectiveness=
Dinesen (2012) [32]	Denmark	Readmissions, costs	-	COPD GOLD stage III–IV	4 m/10 m follow-up	60	51	High	Remote TM	PFTs, HR, SPO2, BP, weight	Hospital readmissions-; time to first exacerbation + (trend)	Costs-
Jehn (2013) [30]	Germany	FEV1, 6MWT, CAT score, AECOPD	-	COPD GOLD stage II–IV, at least one AECOPD during the previous year	9 m	32	30	High	Remote TM	PFTs, CAT, 6MWT	AECOPD-; hospital stay-; specialist consultations-	PFTs=; CAT-; 6MWT+
Paré (2013) [31]	Canada	ED visits, hospital admissions, length of hospitalization, home visits by nurses and respiratory therapists, and economic viability of the program	-	FEV1 < 45%, at least one hospitalization in the previous year	6 m/6 m follow-up	60	60	Mild	Remote TM patient health status and adherence to therapy plus self-management plus telenursing and doctor on demand	Symptoms and medication consumed	ED visits=; hospital admissions-; length of hospitalization-	Cost-effectiveness-; home visits by nurses-; home visits by respiratory physicians=
Pedone (2013) [22]	Italy	AECOPD, related admissions	-	Patients > 65, COPD GOLD stage II and III	9 m	50	49	Mild	Remote TM	HR, SPO2, TEMP, overall physical activity	AECOPD-, hospital admissions—(not statistically significant); length of hospitalization+	-
Segrelles Calvo (2014) [23]	Spain	ED visits, hospital admissions, length of hospitalization, mortality	-	COPD GOLD stage III–IV and LTOT	7 m	30	30	High	Telemonitoring plus teleconsultation plus home visits	PEF, SPO2, HR, BP	ED visits-; hospital admissions-; length of hospitalization-; mortality-	Satisfaction+
Tabak (2014) [33]	Netherlands	Hospital admissions, length of hospitalization, and ED visits	Functional capacity, HRQL, daily physical activity	≥3 AECOPDs or 1 hospitalization for respiratory problems in the 2 years preceding study entry	9 m	15	14	High	Exercising plus self-management plus teleconsultation	-	Hospital admissions-; length of hospitalization-	HRQL+; 6MWT=; satisfaction+
Ho (2016) [19]	Taiwan	Time to first readmission for AECOPD	Time to first ER visit for AECOPD, number of all-cause hospital readmissions, number of all-cause ER visits	Discharge after AECOPD	2 m/6 m follow-up	53	53	Mild	Remote TM, e-diary	SPO2, HR, BP, symptoms, TEMP, weight	Time to first readmission for AECOPD+; time to first ER visit for AECOPD+	Number of all-cause hospital re-admissions-; the number of all-cause ER visits-
Shany (2017) [24]	Australia	ED visits, hospital admissions, length of hospitalization	HRQL, anxiety, depression, costs	COPD GOLD stage III–IV	12 m	21	21	High	Telemonitoring plus e-questionnaire plus telephone support and home visits	PFTs, SPO2, HR, TEMP, BP, ECG, weight, symptoms	ED visits=; hospital admissions=; length of hospitalization-; TTFH+	HRQL=; HADS=; costs-
Vasilopoulou (2017) [34]	Greece	Rate of moderate to severe AECOPDs, hospital admissions, ED visits	Functional capacity, HRQL, daily physical activity	GOLD COPD stage II–IV, and a history of acute exacerbations of COPD 1 year prior to entering the study	2 m/12 m	47	50/50	High	TM plus self-management plus phone contact	SPO2, HR, PFTs, 6MWD, questionnaire	AECOPDs-; hospital admissions-, ED visits-	HRQL+; 6MWT-; SGRQ-; CAT-; mMRC-
Kessler (2018) [20]	France, Germany, Italy, Spain	Length of hospitalization	Number of AECOPDs, acute care hospitalizations, mortality, 6MWT, BODE, HADS, SGQR	COPD GOLD stage III–IV and at least one severe exacerbation in the previous year	12 m	157	162	High	TM plus self-management plus phone contact	PFTs, HR, SPO2, questionnaire plus for patients on LTOT daily oxygen use and RR	Length of hospitalization=; AECOPDs=; acute care hospitalizations-; mortality-; hospital admissions=	BODE-; 6MWT=; SGRQ=; HADS=; quit smoking+
Sink (2020) [25]	USA	TTFH	Hospital admissions	COPD GOLD stage I–IV	8 m	83	85	Low	E-questionnaire plus teleconsultation	Symptoms	TTFH+; hospital admissions-	
Clemente (2021) [27]	Spain	Time to first exacerbation	Number of exacerbations, use of healthcare resources, satisfaction, HRQL, anxiety–depression, therapeutic adherence	Early discharge after AECOPD	7 d/6 m follow-up	58	58	Mild	Remote TM	ECG (leads I, II and III), SPO2, HR, BP, TEMP, and RR	Time to first exacerbation=; number of exacerbations=; costs = (non-inferiority proven)	Use of healthcare resources-; satisfaction+; quality of life+; anxiety-depression=; therapeutic adherence=

BODE: body mass index, airflow obstruction, dyspnea, and exercise capacity; CAT: COPD Assessment Tool; CSQ8: Client Satisfaction Questionnaire-8; COPD: chronic obstructive pulmonary disease; ECG: electrocardiogram; ED: emergency department; FEV1: forced expiratory volume in one second; FOT: forced oscillation technique; HRQL: health-related quality of life; HR: heart rate; HMV: home mechanical ventilation; HADS: hospital anxiety and depression scale; LTOT: long-term oxygen therapy; PEF: peak expiratory flow; PHQ-9: Patient Health Questionnaire; PFTs: pulmonary function tests; RR: respiration rate; SGRQ: Saint George’s Respiratory Questionnaire; SPO2: pulse arterial oxygen saturation; SES: COPD Self-Efficacy Scale; TEMP: temperature; TH: telehealth; TM: telemonitoring; TTFH: time to first hospitalization; 6MWT: six minute walking test.

**Table 2:** Telemedicine studies showing negative results.

Author (Year)	Country	Primary Objective	Secondary Objectives	COPD Severity	TM Duration	*n* Telemonitoring Group	*n* Control Group	Patient Effort Required	Telemonitoring Intervention	Telemonitoring Data	Exacerbation Outcomes	Other Study Outcomes
Lewis (2010) [51]	UK	Hospital admissions	ED visits, length of hospital admissions, GP contacts	GOLD COPD stage II–III	6 m	20	20	Mild	Telemonitoring plus e-questionnaire plus physician on demand	SPO2, TEMP, questionnaire	ED visits=; hospital admissions=; length of hospitalization=; GP contacts for chest problems-	-
Antoniades (2012) [35]	Australia	Hospital admissions, inpatient-days, HRQL	6MWT at baseline and 12 months, adherence to daily monitoring, reproducibility of the physiological measurements, and patient acceptance of RM	GOLD COPD stage II–III, at least 1 hospitalization in the last 12 m	12 m	22	22	High	Remote TM	PFTs, HR, SPO2, BP, TEMP, weight, sputum, symptoms, medication usage	Hospital admissions=; length of hospitalization=	HRQL=; 6MWT=; adherence 80%
Chau (2012) [37]	Hong Kong	Hospital readmissions, use of ED, pulmonary function, user satisfaction, HRQL	-	GOLD COPD stage II–III, at least 1 hospitalization in the last 12 m	2 m	22	18	Mild	Remote TM	SPO2, HR, RR	Hospital readmissions=; use of ED services=	User satisfaction+; HRQL=; pulmonary function=
De San Miguel (2013) [39]	Australia	ED visits, hospital admissions, hospitalization days	Costs, HRQL, satisfaction	Domiciliary oxygen	6 m	40	40	Mild	Remote TM	BP, weight, TEMP, HR, SPO2, questionnaire	ED visits=; hospital admissions=; hospitalization days=	Costs-; HRQL=, over time+; satisfaction+
Jodar-Sanchez (2013) [41]	Spain	ED visits, hospital admissions, HRQL	-	COPD GOLD stage IV, with LTOT, at least one hospitalization for respiratory illness in the previous year	4m	24	21	High	Remote TM	PFTs, HR, SPO2, BP	ED visits=; specialist consultations=; hospital admissions=	HRQL=
Pinnock (2013) [43]	UK	TTFH	TTFH or all cause death, number and duration of hospital admissions, number of deaths at one year, number of exacerbations self-reported by participants, HRQL, anxiety and depression, number and duration of contacts with community services	Patients hospitalized for an AECOPD within the past year in the previous year	12 m	128	128	Mild	Remote telemonitoring, e-diary, telenursing and physician on demand	SPO2, symptoms	TTFH=; TTFH with an AECOPD or all cause death=; number and duration of hospital admissions with an AECOPD=, number and duration of admissions for any cause=; number of deaths at one year-; number of exacerbations self-reported by participants=	HRQL=; HADS=; number and duration of contacts with community services+
Sorknaes (2013) [53]	Denmark	Hospitals readmissions	Mortality, time to mortality and time before first readmission, hospital readmissions per patient, and hospital days per patient	COPD GOLD stage I–IV, hospitalization for AECOPD	7 d/6 m follow-up	132	134	High	Telemonitoring plus teleconsultation	PFTs, SPO2, HR	total hospital readmissions=; time to first readmission=; mortality=; time to mortality=; hospital readmissions per patient=; hospital days per patient=	-
Bentley (2014) [49]	UK	% participants readmitted to hospital with COPD, change in HRQL	% of patients requiring unscheduled healthcare support, cost-effectiveness	Between 1 and 3 admissions in the previous 12 M	2 m TM/6 m follow-up	32	31	Mild	Remote TM	SPO2, HR, BP, symptoms	hospital readmissions+	SGQR+; costs+
Jakobsen (2015) [40]	Denmark	Readmission within 30 days after initial discharge	Mortality, need formanual or mechanical ventilation or NIMV, physiological measures, length of hospitalization, HRQL, user satisfaction, adverse events	COPD GOLD stage III–IV, had an AECOPD and who had an expected hospitalization >2 d	Intervention during home hospitalisation, 6 m follow-up	29	28	High	TM with virtual ward rounds	PFTs, HR, SPO2, TEMP, medicine administration	Non-inferiority not proven	Physiological measures=; length of hospitalization=; HRQL=
McDowell (2015) [42]	Northern Ireland	HRQL	AECOPDs, hospital admissions, ED visits, GP contacts, satisfaction, and cost-effectiveness	GOLD COPD stage II–III, and at least two of: emergency department admissions, hospital admissions or emergency GP contacts in the 12 months before the study	6 m	55	55	Mild	Telemonitoring plus telenursing and physician on demand	BP, HR, SPO2, questionnaire	AECOPDs=; hospital admissions=; ED visits=; GP contacts =	SGRQ-; HADS anxiety score-; HADS depression score=; cost-effectiveness=
Ringbaek (2015) [45]	Denmark	Hospital admissions for AECOPD	Number of all-cause hospital admissions, time to first hospital admission, time to first hospital admission caused by AECOPD, number of ED visits, number of visits to the outpatient clinic, number of AECOPD requiring treatment with systemic steroids or antibiotics but not admission to hospital, length of hospitalization, and all-cause mortality	COPD GOLD stage III–IV, hospital admission due to AECOPD within the previous 36 months and/or treated with LTOT for at least 3 months	6 m	141	140	High	Telemonitoring plus teleconsultation	PFTs, SPO2, mMRC dyspnea scale, sputum color, volume, and purulence	Hospital admissions=; AECOPDs=; all-cause hospital admissions=; time to first hospital admission=; number of ED visits=; length of hospitalization=; number of visits to the outpatient clinic-; number of AECOPD requiring treatment with systemic steroids and/or antibiotics but not admission to hospital+; all-cause mortality=	-
Cordova (2016) [38]	USA	Composite outcome of the number of hospitalizations and deaths	Frequency and severity of AECOPD symptoms, daily PEF, dyspnea score, Duke Activity Status Index, HRQL	Patients hospitalized for an AECOPD within the past year or using supplemental O_2_	24 m	39	40	High	TM plus self-assessment plus phone contact	PEF, dyspnea, sputum quantity, color, and consistency, cough, wheeze, sore throat, nasal congestion, TEMP	Hospital admissions=; length of hospitalization=; AECOPD symptoms-	HRQL=
Vianello (2016) [47]	Italy	HRQL	Number and duration of hospitalizations due to AECOPD, number of readmissions due to AECOPD, number of appointments with a pulmonary specialist, number of ED visits, number of deaths, emotional distress	COPD GOLD stage III–IV	12 m	211	104	Low	TM plus telenursing or nurse and doctor on demand	SPO2, HR	hospitalizations=; length of hospitalization=; readmission rate due to AECOPD-; specialist visits-; ED visits=; deaths=	HRQL=; HADS=
Farmer (2017) [50]	UK	HRQL	Mortality, number with at least one admission, number of AECOPDs, medication adherence, smoking cessation, HRQL, change in lung function, number of GP contacts, number of nurse contacts	COPD GOLD stage II–IV	12 m	110	56	Mild	Remote TM	HR, SPO2, symptoms and anxiety/depression questionnaire	Hospital admissions=; AECOPDs=; mortality=	HRQL=; medication adherence=; smoking cessation=; change in lung function=; number of GP contacts=; number of nurse contacts-
Rose (2018) [44]	Canada	ED visits for AECOPD	Hospitalizations, number of hospitalized days at 1 year, mortality, time to first ED presentation, change in BODE index, HRQL, HADS, COPD Self-Efficacy Scale, Client Satisfaction Questionnaire-8 (CSQ8) and Caregiver Impact Scale	≥1 ED visit or hospital admission for AECOPD in the previous 12 months and ≥2 prognostically-important COPD associated comorbidities	12 m	236	234	Mild	Telehealth plus self-management	Health behavior, symptom monitoring	ED visits=; time to first ED visit=; risk for ED visit-; hospitalizations=; risk for hospital admission-; length of hospitalization-; mortality-;	BODE=; HRQL=; HADS=
Soriano (2018) [46]	Spain	Number of AECOPDs, ED visits, hospital admissions, length of hospitalization	Costs, HRQL, satisfaction	COPD GOLD stage III–IV, LTOT, ≥2 moderate or severe AECOPDs in the previous year (with or without hospitalization)	12 m	115	114	High	TM plus self-management plus teleconsultation	PFTs, SPO2, HR, BP, RR, oxygen therapy compliance	AECOPDs=; ED visits=; hospital admissions=; mortality=; length of hospitalization=; days in ICU=	HRQL=; costs=
Walker (2018) [48]	Spain, United Kingdom, Slovenia, Estonia, and Sweden	TTFH, HRQL	Moderate exacerbation rate;hospitalizations; CAT, PHQ-9, and MLHFQ questionnaires; and cost–utility analysis	COPD GOLD stage ≥ II and a history of AECOPD in the previous 12 months	9 m	154	158	High	Remote TM	within-breath respiratory mechanical impedance using FOT	TTFH=; hospitalizations=; moderate exacerbations=; readmission rate due to AECOPD-	HRQL=
Boer (2019) [36]	Netherlands	Exacerbation-free time	Exacerbation-related outcomes, health status, self-efficacy, self-management behavior, healthcare utilization, and usability	≥2 AECOPDs in the previous 12 months	12 m	43	44	High	Self-management with an innovative mobile health tool	PFTs, HR, SPO2, TEMP, questionnaire concerning changes in symptoms, physical limitations, and emotions	exacerbation-free weeks=	health status=; self-efficacy=; self-management behavior=; healthcare utilization=
Rassouli (2021) [52]	Switzerland and Germany	Difference in weekly CAT score	Number of AECOPDs and hospital admissions, length of hospitalization, treatment costs per patient and year	FEV1 51%	12 m	84	84	Mild	Telehealth plus self-management	Daily symptoms, CAT score	AECOPDs=; ED visits=; hospital admissions=; length of hospitalization=	CAT score-; satisfaction+; Costs=

BODE: body mass index, airflow obstruction, dyspnea, and exercise capacity; CAT: COPD Assessment Tool; CSQ8: Client Satisfaction Questionnaire-8; COPD: chronic obstructive pulmonary disease; ECG: electrocardiogram; ED: emergency department; FEV1: forced expiratory volume in one second; FOT: forced oscillation technique; HRQL: health-related quality of life; HR: heart rate; HMV: home mechanical ventilation; HADS: hospital anxiety and depression scale; LTOT: long term oxygen therapy; PEF: peak expiratory flow; PHQ-9: Patient Health Questionnaire; PFTs: pulmonary function tests; RR: respiration rate; SGRQ: Saint George’s Respiratory Questionnaire; SPO2: pulse arterial oxygen saturation; SES: COPD Self-Efficacy Scale; TEMP: temperature; TH: telehealth; TM: telemonitoring; TTFH: time to first hospitalization; 6MWT: six minute walking test.

**Table 3:** Proposed characteristics of future studies.

A minimum 12-month follow-up period
AECOPDs and healthcare utilization should be the primary endpoint
Appropriate parameters monitored in terms of reliable prediction of an AECOPD (e.g., FEV1, symptoms, pulse oximetry, heart rate, respiratory rate)
Large number of participants
Subgroup analysis in order to define the most appropriate population for telemonitoring intervention
Commitment and appropriate training of the study team involved
Telemonitoring and teleconsultation during the weekends
Interventions that would require minimal effort on the patients’ end would achieve higher compliance

## Data Availability

All data generated or analyzed during this study are included in this published article. Anonymized data will be shared uponrequest from any qualified investigator.

## Abbreviations

BODE	body mass index, airflow obstruction, dyspnea, and exercise capacity
CAT	COPD Assessment Tool
CSQ8	Client Satisfaction Questionnaire-8
COPD	Chronic Obstructive Pulmonary Disease
ECG	electrocardiogram
ED	emergency department
FEV1	forced expiratory volume in one second
FOT	forced oscillation technique
GP	general practitioner
HRQL	health-related quality of life
HR	heart rate
HMV	home mechanical ventilation
HADS	hospital anxiety and depression scale
LTOT	long term oxygen therapy
PEF	peak expiratory flow
PHQ-9	Patient Health Questionnaire
PFTs	pulmonary function tests
RR	respiratory rate
SES	COPD Self-Efficacy Scale
SGRQ	Saint George’s Respiratory Questionnaire
SOC	standard of care
SPO2	pulse arterial oxygen saturation
TEMP	temperature
TH	telehealth
TM	telemonitoring
TTFH	time to first hospitalization

## References

[1] Adeloye D., Chua S., Lee C., Basquill C., Papana A., Theodoratou E., Nair H., Gasevic D., Sridhar D., Campbell H. (2015). Global and Regional Estimates of COPD Prevalence: Systematic Review and Meta-Analysis. J. Glob. Health.

[2] World Health Organization (2020). https://www.who.int/news-room/fact-sheets/detail/the-top-10-causes-of-death.

[3] 2021 GOLD Reports. https://goldcopd.org/2021-gold-reports.

[4] Galani M., Kyriakoudi A., Filiou E., Kompoti M., Lazos G., Gennimata S., Vasileiadis I., Daganou M., Koutsoukou A., Rovina N. (2021). Older Age, Disease Severity and Co-Morbidities Independently Predict Mortality in Critically Ill Patients with COPD Exacerbation. Pneumon.

[5] Anzueto A. (2010). Impact of Exacerbations on COPD. Eur. Respir. Rev..

[6] Papaioannou A., Bartziokas K., Loukides S., Papiris S., Kostikas K. (2016). “Get Well Soon!” Why Fast Recovery from a COPD Exacerbation Matters. Pneumon.

[7] Vitacca M., Montini A., Comini L. (2018). How Will Telemedicine Change Clinical Practice in Chronic Obstructive Pulmonary Disease?. Ther. Adv. Respir. Dis..

[8] Strehle E.M., Shabde N. (2006). One Hundred Years of Telemedicine: Does This New Technology Have a Place in Paediatrics?. Arch. Dis. Child..

[9] Hjelm N.M., Julius H.W. (2005). Centenary of Tele-Electrocardiography and Telephonocardiography. J. Telemed. Telecare.

[10] Gaveikaite V., Fischer C., Schonenberg H., Pauws S., Kitsiou S., Chouvarda I., Maglaveras N., Roca J. (2018). Telehealth for Patients with Chronic Obstructive Pulmonary Disease (COPD): A Systematic Review and Meta-Analysis Protocol. BMJ Open.

[11] Bourbeau J., Farias R. (2018). Making Sense of Telemedicine in the Management of COPD. Eur. Respir. J..

[12] Polisena J., Tran K., Cimon K., Hutton B., McGill S., Palmer K., Scott R.E. (2010). Home Telehealth for Chronic Obstructive Pulmonary Disease: A Systematic Review and Meta-Analysis. J. Telemed. Telecare.

[13] Lundell S., Holmner Å., Rehn B., Nyberg A., Wadell K. (2015). Telehealthcare in COPD: A Systematic Review and Meta-Analysis on Physical Outcomes and Dyspnea. Respir. Med..

[14] McLean S., Nurmatov U., Liu J.L., Pagliari C., Car J., Sheikh A. (2011). Telehealthcare for Chronic Obstructive Pulmonary Disease. Cochrane Database Syst. Rev..

[15] Pedone C., Lelli D. (2015). Systematic Review of Telemonitoring in COPD: An Update. Pneumonol. I Alergol. Pol..

[16] Kitsiou S., Paré G., Jaana M. (2013). Systematic Reviews and Meta-Analyses of Home Telemonitoring Interventions for Patients with Chronic Diseases: A Critical Assessment of Their Methodological Quality. J. Med. Internet Res..

[17] Barbosa M.T., Sousa C.S., Morais-Almeida M., Simões M.J., Mendes P. (2020). Telemedicine in COPD: An Overview by Topics. COPD.

[18] Jang S., Kim Y., Cho W.-K. (2021). A Systematic Review and Meta-Analysis of Telemonitoring Interventions on Severe COPD Exacerbations. Int. J. Environ. Res. Public Health.

[19] Ho T.-W., Huang C.-T., Chiu H.-C., Ruan S.-Y., Tsai Y.-J., Yu C.-J., Lai F. (2016). HINT Study Group Effectiveness of Telemonitoring in Patients with Chronic Obstructive Pulmonary Disease in Taiwan—A Randomized Controlled Trial. Sci. Rep..

[20] Kessler R., Casan-Clara P., Koehler D., Tognella S., Viejo J.L., Dal Negro R.W., Díaz-Lobato S., Reissig K., Rodríguez González-Moro J.M., Devouassoux G. (2018). COMET: A Multicomponent Home-Based Disease-Management Programme Routine Care in Severe COPD. Eur. Respir. J..

[21] Koff P.B., Jones R.H., Cashman J.M., Voelkel N.F., Vandivier R.W. (2009). Proactive Integrated Care Improves Quality of Life in Patients with COPD. Eur. Respir. J..

[22] Pedone C., Chiurco D., Scarlata S., Incalzi R.A. (2013). Efficacy of MultiparametricTelemonitoring on Respiratory Outcomes in Elderly People with COPD: A Randomized Controlled Trial. BMC Health Serv. Res..

[23] SegrellesCalvo G., Gómez-Suárez C., Soriano J.B., Zamora E., Gónzalez-Gamarra A., González-Béjar M., Jordán A., Tadeo E., Sebastián A., Fernández G. (2014). A Home Telehealth Program for Patients with Severe COPD: The PROMETE Study. Respir. Med..

[24] Shany T., Hession M., Pryce D., Roberts M., Basilakis J., Redmond S., Lovell N., Schreier G. (2017). A Small-Scale Randomised Controlled Trial of Home Telemonitoring in Patients with Severe Chronic Obstructive Pulmonary Disease. J. Telemed. Telecare.

[25] Sink E., Patel K., Groenendyk J., Peters R., Som A., Kim E., Xing M., Blanchard M., Ross W. (2020). Effectiveness of a Novel, Automated Telephone Intervention on Time to Hospitalisation in Patients with COPD: A Randomised Controlled Trial. J. Telemed. Telecare.

[26] Vitacca M., Bianchi L., Guerra A., Fracchia C., Spanevello A., Balbi B., Scalvini S. (2009). Tele-Assistance in Chronic Respiratory Failure Patients: A Randomised Clinical Trial. Eur. Respir. J..

[27] Mínguez Clemente P., Pascual-Carrasco M., Mata Hernández C., Malo de Molina R., Arvelo L.A., Cadavid B., López F., Sánchez-Madariaga R., Sam A., Trisan Alonso A. (2021). Follow-up with Telemedicine in Early Discharge for COPD Exacerbations: Randomized Clinical Trial (TELEMEDCOPD-Trial). COPD.

[28] De Toledo P., Jiménez S., del Pozo F., Roca J., Alonso A., Hernandez C. (2006). Telemedicine Experience for Chronic Care in COPD. IEEE Trans. Inf. Technol. Biomed..

[29] Casas A., Troosters T., Garcia-Aymerich J., Roca J., Hernández C., Alonso A., del Pozo F., de Toledo P., Antó J.M., Rodríguez-Roisín R. (2006). Integrated Care Prevents Hospitalisations for Exacerbations in COPD Patients. Eur. Respir. J..

[30] Jehn M., Donaldson G., Kiran B., Liebers U., Mueller K., Scherer D., Endlicher W., Witt C. (2013). Tele-Monitoring Reduces Exacerbation of COPD in the Context of Climate Change—A Randomized Controlled Trial. Environ. Health.

[31] Paré G., Poba-Nzaou P., Sicotte C., Beaupré A., Lefrançois É., Nault D., Saint-Jules D. (2013). Comparing the Costs of Home Telemonitoring and Usual Care of Chronic Obstructive Pulmonary Disease Patients: A Randomized Controlled Trial. Eur. Res. Telemed..

[32] Dinesen B., Haesum L.K.E., Soerensen N., Nielsen C., Grann O., Hejlesen O., Toft E., Ehlers L. (2012). Using Preventive Home Monitoring to Reduce Hospital Admission Rates and Reduce Costs: A Case Study of Telehealth among Chronic Obstructive Pulmonary Disease Patients. J. Telemed. Telecare.

[33] Tabak M., Brusse-Keizer M., van der Valk P., Hermens H., Vollenbroek-Hutten M. (2014). A Telehealth Program for Self-Management of COPD Exacerbations and Promotion of an Active Lifestyle: A Pilot Randomized Controlled Trial. Int. J. Chron. Obstruct. Pulmon. Dis..

[34] Vasilopoulou M., Papaioannou A.I., Kaltsakas G., Louvaris Z., Chynkiamis N., Spetsioti S., Kortianou E., Genimata S.A., Palamidas A., Kostikas K. (2017). Home-Based Maintenance Tele-Rehabilitation Reduces the Risk for Acute Exacerbations of COPD, Hospitalisations and Emergency Department Visits. Eur. Respir. J..

[35] Antoniades N.C., Rochford P.D., Pretto J.J., Pierce R.J., Gogler J., Steinkrug J., Sharpe K., McDonald C.F. (2012). Pilot Study of Remote Telemonitoring in COPD. Telemed. J. E. Health.

[36] Boer L., Bischoff E., van der Heijden M., Lucas P., Akkermans R., Vercoulen J., Heijdra Y., Assendelft W., Schermer T. (2019). A Smart Mobile Health Tool Versus a Paper Action Plan to Support Self-Management of Chronic Obstructive Pulmonary Disease Exacerbations: Randomized Controlled Trial. JMIR MhealthUhealth.

[37] Chau J.P.-C., Lee D.T.-F., Yu D.S.-F., Chow A.Y.-M., Yu W.-C., Chair S.-Y., Lai A.S.F., Chick Y.-L. (2012). A Feasibility Study to Investigate the Acceptability and Potential Effectiveness of a Telecare Service for Older People with Chronic Obstructive Pulmonary Disease. Int. J. Med. Inform..

[38] Cordova F.C., Ciccolella D., Grabianowski C., Gaughan J., Brennan K., Goldstein F., Jacobs M.R., Criner G.J. (2016). A Telemedicine-Based Intervention Reduces the Frequency and Severity of COPD Exacerbation Symptoms: A Randomized, Controlled Trial. Telemed. J. E. Health.

[39] De San Miguel K., Smith J., Lewin G. (2013). Telehealth Remote Monitoring for Community-Dwelling Older Adults with Chronic Obstructive Pulmonary Disease. Telemed. J. E. Health.

[40] Jakobsen A.S., Laursen L.C., Rydahl-Hansen S., Østergaard B., Gerds T.A., Emme C., Schou L., Phanareth K. (2015). Home-Based Telehealth Hospitalization for Exacerbation of Chronic Obstructive Pulmonary Disease: Findings from “the Virtual Hospital” Trial. Telemed. J. E Health.

[41] Jódar-Sánchez F., Ortega F., Parra C., Gómez-Suárez C., Jordán A., Pérez P., Bonachela P., Leal S., Barrot E. (2013). Implementation of a TelehealthProgramme for Patients with Severe Chronic Obstructive Pulmonary Disease Treated with Long-Term Oxygen Therapy. J. Telemed. Telecare.

[42] McDowell J.E., McClean S., FitzGibbon F., Tate S. (2015). A Randomised Clinical Trial of the Effectiveness of Home-Based Health Care with Telemonitoring in Patients with COPD. J. Telemed. Telecare.

[43] Pinnock H., Hanley J., McCloughan L., Todd A., Krishan A., Lewis S., Stoddart A., van der Pol M., MacNee W., Sheikh A. (2013). Effectiveness of Telemonitoring Integrated into Existing Clinical Services on Hospital Admission for Exacerbation of Chronic Obstructive Pulmonary Disease: Researcher Blind, Multicentre, Randomised Controlled Trial. BMJ.

[44] Rose L., Istanboulian L., Carriere L., Thomas A., Lee H.-B., Rezaie S., Shafai R., Fraser I. (2018). Program of Integrated Care for Patients with Chronic Obstructive Pulmonary Disease and Multiple Comorbidities (PIC COPD): A Randomised Controlled Trial. Eur. Respir. J..

[45] Ringbæk T., Green A., Laursen L.C., Frausing E., Brøndum E., Ulrik C.S. (2015). Effect of Tele Health Care on Exacerbations and Hospital Admissions in Patients with Chronic Obstructive Pulmonary Disease: A Randomized Clinical Trial. Int. J. Chron. Obstruct. Pulmon. Dis..

[46] Soriano J.B., García-Río F., Vázquez-Espinosa E., Conforto J.I., Hernando-Sanz A., López-Yepes L., Galera-Martínez R., Peces-Barba G., Gotera-Rivera C.M., Pérez-Warnisher M.T. (2018). A Multicentre, Randomized Controlled Trial of Telehealth for the Management of COPD. Respir. Med..

[47] Vianello A., Fusello M., Gubian L., Rinaldo C., Dario C., Concas A., Saccavini C., Battistella L., Pellizzon G., Zanardi G. (2016). Home Telemonitoring for Patients with Acute Exacerbation of Chronic Obstructive Pulmonary Disease: A Randomized Controlled Trial. BMC Pulm. Med..

[48] Walker P.P., Pompilio P.P., Zanaboni P., Bergmo T.S., Prikk K., Malinovschi A., Montserrat J.M., Middlemass J., Šonc S., Munaro G. (2018). Telemonitoring in Chronic Obstructive Pulmonary Disease (CHROMED). A Randomized Clinical Trial. Am. J. Respir. Crit. Care Med..

[49] Bentley C.L., Mountain G.A., Thompson J., Fitzsimmons D.A., Lowrie K., Parker S.G., Hawley M.S. (2014). A Pilot Randomised Controlled Trial of a Telehealth Intervention in Patients with Chronic Obstructive Pulmonary Disease: Challenges of Clinician-Led Data Collection. Trials.

[50] Farmer A., Williams V., Velardo C., Shah S.A., Yu L.-M., Rutter H., Jones L., Williams N., Heneghan C., Price J. (2017). Self-Management Support Using a Digital Health System Compared With Usual Care for Chronic Obstructive Pulmonary Disease: Randomized Controlled Trial. J. Med. Internet Res..

[51] Lewis K.E., Annandale J.A., Warm D.L., Rees S.E., Hurlin C., Blyth H., Syed Y., Lewis L. (2010). Does Home Telemonitoring after Pulmonary Rehabilitation Reduce Healthcare Use in Optimized COPD? A Pilot Randomized Trial. COPD.

[52] Rassouli F., Germann A., Baty F., Kohler M., Stolz D., Thurnheer R., Brack T., Kähler C., Widmer S., Tschirren U. (2021). Telehealth Mitigates COPD Disease Progression Compared to Standard of Care: A Randomized Controlled Crossover Trial. J. Intern. Med..

[53] Sorknaes A.D., Bech M., Madsen H., Titlestad I.L., Hounsgaard L., Hansen-Nord M., Jest P., Olesen F., Lauridsen J., Østergaard B. (2013). The Effect of Real-Time Teleconsultations between Hospital-Based Nurses and Patients with Severe COPD Discharged after an Exacerbation. J. Telemed. Telecare.

